# Vaccinology in the twenty-first century

**DOI:** 10.1038/npjvaccines.2016.9

**Published:** 2016-07-28

**Authors:** Alan D T Barrett

**Affiliations:** 1 Sealy Center for Vaccine Development, University of Texas Medical Branch, Galveston, TX, USA; 2 Department of Pathology, University of Texas Medical Branch, Galveston, TX, USA

Vaccinology is defined as the science of vaccines, and historically includes basic science, immunogens, the host immune response, delivery strategies and technologies, manufacturing, and clinical evaluation. More recently, the science has expanded further to include the safety, regulatory, ethical and economic considerations of vaccine development and utilisation. Veterinary vaccines are equally important in the field of vaccinology for their contribution not only to animal health but also to the security of the food supply for humans. Although traditionally vaccinology has focused on infectious diseases, as we move forward in the twenty-first century vaccines will also potentially make significant contributions to the control of non-infectious diseases such as cancers, neurodegenerative diseases and addictions.

*npj Vaccines* is a new open access, online-only, journal, which is part of the Nature Research portfolio and published in partnership with the University of Texas Medical Branch, Sealy Center for Vaccine Development. The journal is dedicated to highlighting the most important scientific advances in vaccine research and development. Given the public health importance of vaccines, in addition to publishing high-quality original research, *npj Vaccines* will also publish reviews, perspectives, commentaries, editorials and correspondence from readers, to provide the most current information and developments for those interested in vaccines.

## Vaccines in the twentieth century

It seems as though centuries have passed since we routinely experienced childhood diseases such as polio, measles, diphtheria, *Haemophilus influenzae* type B and rubella, and much of society cannot remember the often devastating impact of these once common infectious diseases. Rarely, if ever, do we see someone on crutches as a result of paralytic poliomyelitis or with a hearing deficiency caused by a measles infection. We owe a huge debt of gratitude to the pioneering vaccinologists of the twentieth century for their success in developing vaccines for many of the life-threatening childhood diseases, success that resulted in the US Centers for Disease Control and Prevention considering vaccines the greatest public health achievement of the twentieth century.^1^ Similarly, there have been comparable successes with the development of many veterinary vaccines and to date the crowning glories of the science of vaccinology are the eradication of smallpox for humans and rinderpest for cattle.^2,3^

## Vaccine research and development in the twenty-first century

Vaccine research and development has changed greatly over the past 30 years from an empirical approach to one that is rational and supported by both basic and applied science. Examples that come to mind are the hepatitis B vaccine developed by expression of hepatitis B surface antigen in yeast,^4^ the meningococcal type B vaccine developed by reverse vaccinology^5^ and the tetravalent live-attenuated dengue vaccine developed using reverse genetics.^6^ Concurrently, there has been a continual increase in the quantity and quality of data needed to support the vaccine development pathway for vaccine licensure. Thus, not only have we seen a move by vaccine developers to exploit new technologies but regulatory science is also utilising this information for their evaluation of the safety and efficacy of candidate vaccines. Overall, this has had positive and negative effects. Positive is that we know far more about the characteristics of any particular vaccine and safety has improved. However, the bad news is that we have not seen a corresponding increase in the number of vaccines licensed. This increased knowledge and better understanding appears to have lengthened the time taken to generate the required data and information required for licensure. This situation is unlikely to change in the foreseeable future.

## How many vaccines are licensed and who pays?

I always ask students in my vaccinology classes how many human vaccines are there. Usually, the answer varies between 100 and 1,000. The students are always surprised when I tell them that there are ~50 human vaccines, of which only 30–40 are licensed in the United States and Europe. There have actually been more veterinary vaccinology success stories with over 300 vaccines licensed around the world. Although this demonstrates our success in developing both human and veterinary vaccines, it also emphasises that, in general, society does not understand the ongoing need to continue developing new vaccines, or the necessity of providing resources for the research and development required for those additional vaccines. This situation is made even more difficult by society’s expectations that a vaccine must be 100% effective, 150% safe and cost US$1 (or less for veterinary vaccines). Clearly, an impossible task!

As the twenty-first century progresses we need to come to terms with who will pay for vaccine research and development. Every vaccine in development involves molecular techniques, and regulators continue to expand utilisation of these techniques for safety and quality control requirements. As vaccine research and development continues to become more sophisticated with its use of state-of-the-art molecular techniques, so do the costs. It is speculated that Sanofi Pasteur’s recently licensed recombinant, chimeric, tetravalent live-attenuated dengue vaccine took 24 years and US$1.6 billion to develop. Our current model is that pharmaceutical companies will recoup their costs via the price of the product, yet the consumer is often not prepared to pay those costs unless they feel personally threatened by a particular infectious disease. In recent years, a few governments and some non-governmental organisations have assumed these costs, and there is no doubt that some vaccines would not have been developed without their support; the development and testing of Ebola vaccines in 2014–2015 is one that immediately comes to mind.^7^

But it is not just a case of ‘research and development’ of a vaccine, there is also the question of educating people to take a vaccine, particularly new ones. Vaccine implementation has become an increasingly important area such that we should probably not think in terms of the ‘vaccine research and development pathway’ but the ‘vaccine research, development and implementation pathway’.

## Vaccines for emerging diseases

The twenty-first century has demonstrated that we can use molecular techniques to acquire detailed knowledge of a previously unknown, infectious agent in record time, as exemplified by severe acute respiratory syndrome coronavirus and Middle East respiratory syndrome coronavirus, and can then use this information for the development of candidate vaccines. The recent 2014–2015 Ebola epidemic in Africa demonstrated how 10 years of preclinical development can be rapidly translated into clinical evaluation with the joint efforts of funders, developers and regulators. It is no accident that the leading vaccine candidates were developed using molecular techniques with three live-vectored vaccines (recombinant vesicular stomatitis virus, recombinant-modified vaccinia Ankara and recombinant adenovirus) and recombinant subunit glycoprotein vaccines. These are all ‘platform’ technologies, and demonstrate how far vaccinology has progressed from ‘one bug-one vaccine’ to generic vaccine platforms for multiple candidate vaccines. Despite having come a long way in a very short period of time, we are yet to have a licensed human vaccine for any of these emerging diseases, although there are examples of licensed veterinary emerging disease vaccines, recombinant subunit protein vaccine developed for Hendra in Australia and recombinant vaccines for West Nile in the United States and Europe. Why are there no licensed human emerging disease vaccines? This is in part due to the transient nature of emerging diseases. The occurrence of these previously unknown public health threats prompts immediate responses from government and non-government funders; huge resources are allocated and immense efforts by academia, pharmaceutical companies, government and non-government scientists occur in a very short period of time. The good news is that we control the emerging disease; the bad news is that our successes result in loss of interest by the media and those who control funding resources as other priorities overtake the emerging disease. In this respect, Ebola in 2014–2015 has been replaced by Zika in 2015–2016. The progress on this emerging disease has been nothing short of amazing. As the first published report on Zika in May 2015, there have been over 700 papers in PubMed plus many others on non-peer-reviewed servers. From a ‘ground zero’ where little was known about the virus or disease, there are now mouse and non-human primate models for preclinical development, over 40 vaccine candidates in discovery or preclinical development, and the strong likelihood that there will be phase I clinical trials before the end of 2016. Again, this is an excellent example of how academia, pharmaceutical companies, government and non-government scientists, plus regulators and funders are all working together.

There is no doubt that emerging diseases will continue to be public health threats in the future. Somehow, we need to avoid the ‘knee-jerk’ response. One approach would be to create ‘international centres of excellence in emerging disease research’, with guaranteed funding, whose role would be to undertake research and development on vaccines for these diseases. Such centres would need to be public–private partnerships, rather than product development partnerships, as by definition we do not know when an emerging disease will become a public health threat, and vaccine research and development needs not only basic and applied science expertise but also the expertise of pharmaceutical companies for scale-up and manufacturing. As shown by the Ebola epidemic, there is little need for routine vaccination for many emerging diseases, and major efforts are in stockpiling vaccines, which will be replenished at the end of their shelf life if they are not used. This approach is of limited interest to large vaccine pharmaceutical companies due to the low return on investment.

## Personalised vaccines

As we move forward in the twenty-first century, it has also become evident that not all humans or animals respond to vaccines in the same way. Some individuals have strong immune responses, whereas others have weak or even undetectable responses. Similarly, the reactogenicity of a particular vaccine varies at the individual level. Many of these differences are due to the host response to vaccination. Despite learning much about the host innate and adaptive immune response to vaccination at the population level, our understanding is somewhat rudimentary at the individual level, although it is clear that genetic factors have a role in our response to individual vaccines. We have much to learn. The area of ‘systems vaccinology’, including genomics, proteomics, transcriptomics, informatics, etc., will become increasingly important in the future as it bridges between basic and clinical science, and provides information on safety and immunogenicity. Furthermore, this information should eventually enable a particular vaccine to be available as different formulations based on the genetic background of the host. This may not appear economically viable at the present time but should change in the future. Concurrently, the utilisation of appropriate adjuvants and alternative routes of delivery for a particular vaccine provide great opportunities to improve the efficacy of vaccines in the future.

## Non-infectious disease vaccines

As we move into the twenty-first century, infectious disease vaccine research is focused on ‘difficult’ childhood diseases and the application of childhood vaccines to adults and the elderly with the goal of enabling adults to live longer. However, infectious diseases are not the primary cause of morbidity and mortality for adults. The World Health Organization has identified that chronic diseases will be the leading cause of disability worldwide by 2020. Although it is not possible at this time to develop prophylactic vaccines for these diseases, development of therapeutic vaccines will lead to opportunities to manage these diseases at early stages. Examples of such diseases include cancers, obesity, neurodegenerative diseases, addictions and atherosclerosis. Such vaccines would be expected to extend the life expectancy of adults and reduce the burden of disease. At the present time, such research is in the discovery stage, similar to infectious disease vaccine research during the twentieth century. If correct, the golden years of non-infectious disease vaccine research are ahead of us. Much of the focus so far has been on therapeutic administration of antibodies, in particular monoclonal antibodies. This approach is promising and has been very successful for infectious diseases. A notable example would be for respiratory syncytial virus. The development of a third-generation humanised monoclonal antibody where one dose gives protection for 6 months has clear applicability to non-infectious diseases. One surprising aspect of non-infectious disease vaccine research is the apparent lack of collaborations between those with infectious disease vaccine expertise and those working on non-infectious disease vaccines. Such interdisciplinary collaborations would be predicted to accelerate non-infectious disease vaccine research and development.

The goal of *npj Vaccines* is to highlight the most important scientific advances in human and veterinary vaccine research and development. It is a multidisciplinary journal that is dedicated to publishing the finest, high-quality research and development on human and veterinary vaccines, including discovery and basic science, nonclinical development of vaccines, clinical evaluation of vaccines, vaccine safety, and regulatory science. Our first articles provide important new information on emerging disease vaccines. Two are on henipaviruses, one a veterinary vaccine for Hendra^8^ and the other a human vaccine for Nipah.^9^ In addition, there is a review on the current status of vaccine development for Zika.^10^ Also, we have a very interesting paper investigating head and stalk antibodies in polyclonal sera obtained from health-care workers following vaccination with a monovalent pandemic H1N1 influenza vaccine and an associated comment.^11,12^ We are encouraged by the high quality of the content of papers received to date, and look forward to your contributions in the future. Happy reading!
